# Near-Field Excitation of Bound States in the Continuum in All-Dielectric Metasurfaces through a Coupled Electric/Magnetic Dipole Model

**DOI:** 10.3390/nano11040998

**Published:** 2021-04-13

**Authors:** Diego R. Abujetas, José A. Sánchez-Gil

**Affiliations:** Instituto de Estructura de la Materia (IEM-CSIC), Consejo Superior de Investigaciones Científicas, Serrano 121, 28006 Madrid, Spain; j.sanchez@csic.es

**Keywords:** coupled dipole theories, all-dielectric metasurfaces, bound states in the continuum, plasmonics, nanophotonics

## Abstract

Resonant optical modes arising in all-dielectric metasurfaces have attracted much attention in recent years, especially when so-called bound states in the continuum (BICs) with diverging lifetimes are supported. With the aim of studying theoretically the emergence of BICs, we extend a coupled electric and magnetic dipole analytical formulation to deal with the proper metasurface Green function for the infinite lattice. Thereby, we show how to excite metasurface BICs, being able to address their near-field pattern through point-source excitation and their local density of states. We apply this formulation to fully characterize symmetry-protected BICs arising in all-dielectric metasurfaces made of Si nanospheres, revealing their near-field pattern and local density of states, and, thus, the mechanisms precluding their radiation into the continuum. This formulation provides, in turn, an insightful and fast tool to characterize BICs (and any other leaky/guided mode) near fields in all-dielectric (and also plasmonic) metasurfaces, which might be especially useful for the design of planar nanophotonic devices based on such resonant modes.

## 1. Introduction

Coupled-dipole formulations have been exploited since long ago to describe the optical properties of particles (typically of sub-wavelength dimensions) behaving as point dipoles [1,2]. These formulations allow to address the coupling between such dipolar particles in a variety of arrangements. In recent years, 2D planar arrays consisting of resonant dipolar/multipolar particles have attracted increased attention [3,4,5,6,7,8,9,10,11,12,13,14,15,16,17,18,19,20], lately including magnetic dipole resonances to account for the lowest-order Mie resonances of high-refractive index particles [5,21,22,23,24,25,26]. The renewed interest in such planar arrays stems from their application as infinitely thin optical devices performing various functionalities [27,28,29,30,31,32,33,34,35,36,37,38,39,40], especially in nanophotonics, known as metasurfaces/metagratings in the non-diffractive/diffractive spectral regime, respectively.

Among the rich phenomenology they exhibit, metasurfaces have been shown to be a suitable platform to support bound states in the continuum (BICs) with diverging Q factors [36,41,42,43,44,45,46,47,48,49]. The infinitely long (theoretical) lifetimes of such exotic states have been exploited to enhance sensing [50,51,52,53], filtering [54], lasing [55,56,57,58,59], electromagnetically-induced transparency [60], chirality [61], and non-linearities [62,63,64], thus holding promise of unprecedented planar devices in nanophotonics.

Interestingly, BICs arising in metasurfaces are typically explored through far-field reflection/transmission; since they are not accessible under (propagating) plane wave illumination by definition, BICs are observed as the vanishing of the quasi-BIC in certain parameter space when the BIC condition is approached (quasi-BICs). Thus, it would be desirable to have means to directly address BICs through resonant mode characterization and near-field excitation, including local density of states (LDOS), rigorously dealing with complex interactions between particle and lattice resonances in planar infinite arrays. This would also shed light onto the various mechanisms precluding BIC radiation into the far field, symmetry protection and accidental degeneracy being particularly relevant to metasurfaces; see, e.g., References [42,45]. Full wave numerical calculations very often lack accuracy and speed when dealing with modes with diverging Q-factor, not to mention the fact that they cannot shed much light onto the underlying physics.

In this work, we extend a previously developed coupled electric and magnetic dipole (CEMD) formulation for plane wave excitation [19], to deal with the proper Green function of the scattering problems. This allows in practice to consider point-dipole near-field excitation, mode dispersion relation, and local density of electromagnetic states. Thereby, we analyze the emergence of BICs in a planar array of silicon nanospheres, characterizing their vectorial LDOS at a plane close to the unit cell. Such LDOS information is used to explore the proper conditions (position and polarization) for point dipole excitation of BICs, in turn revealed through the near-field pattern calculated across the metasurface plane, with symmetry protection being evidenced. Finally, the conclusions extracted from such rich near-field BIC properties are drawn.

## 2. Methods: Coupled Electric/Magnetic Dipole Model for Point Dipole Excitation

**Figure 1:**
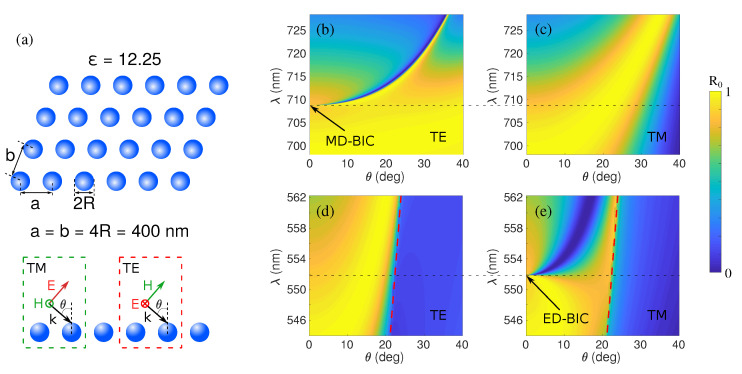
Color maps of the reflectance as a function of wavelength and angle of incidence for both polarizations for a square array of dielectric spheres (radius R=100 nm) of constant dielectric permittivity ϵ=12.25 (n=3.5, similar to that of Si in the visible), with lattice constants a=b=4R=400 nm, as shown in (**a**), in the vicinity of two different bound states in the continuum (BICs) (at wavelengths indicated by black dashed horizontal lines): (**b**,**d**) TE and (**c**,**e**) TM polarizations. The symmetry-protected BICs emerge at: (**b**) MD-BIC for TE polarization at λ=709 nm and (**e**) ED-BIC for TM polarization at λ=552 nm. Dashed red lines in (**d**,**e**) delimit the diffractive regions (right); none in (**b**,**c**).

Let us first consider a monochromatic point dipole source located at at an arbitrary position $r_{d}=\left(x_{d},y_{d},z_{d}\right)$ with respect to a planar array of scatterers located in the plane z=0 (see Figure 1a). Using the Weyl expansion, the field emitted by the dipole at the position r=(x,y,z), $\psi^{d}\left(\omega ,r-r_{d}\right)$ can be expressed as:

(1) $$\begin{array}{c} \psi^{d}\left(\omega ,r-r_{d}\right)=\int \frac{dQ_{x}dQ_{y}}{4\pi^{2}}e^{ıQ_{x}\left(x-x_{d}\right)}e^{ıQ_{y}\left(y-y_{d}\right)}\frac{ı}{2q}e^{ıq|z-z_{d}|}\overset{↔}{M}\left(\omega ,Q_{x},Q_{y}\right)P, \end{array}$$

(2) $$\begin{array}{c} q=\sqrt{k^{2}-Q_{x}^{2}-Q_{y}^{2}}, \end{array}$$

with k=ω/c, where *c* and ω are the speed and frequency of light, P characterizes the polarization of the dipole (both electric and magnetic), and $\overset{↔}{M}\left(\omega ,Q_{x},Q_{y}\right)$ is the matrix Green function of a point (electric and magnetic) dipole.

Thus Equation (1) can be formally written as a sum of (integral over) plane waves:

(3) $$\begin{array}{c} \psi^{d}\left(\omega ,r-r_{d}\right)=\int dQ_{x}dQ_{y}I^{d}\left(\omega ,r_{d},Q_{x},Q_{y}\right)e^{ıQ_{x}x}e^{ıQ_{y}y}e^{\pm ıqz}v^{d}\left(\omega ,Q_{x},Q_{y}\right), \end{array}$$

where the intensity and the vector polarization distribution, $I^{d}$ and $v^{d}$, respectively, are

(4) $$\begin{array}{c} I^{d}\left(\omega ,r_{d},Q_{x},Q_{y}\right)=\frac{1}{4\pi^{2}}e^{-ıQ_{x}x_{d}}e^{-ıQ_{y}y_{d}}\frac{ı}{2q}e^{\mp ıqz_{d}}, \end{array}$$

(5) $$\begin{array}{c} v^{d}=\overset{↔}{M}\left(\omega ,Q_{x},Q_{y}\right)P. \end{array}$$

The sign (±) of the exponentials that depend on *z* and $z_{d}$ is chosen depending on the sign of $\left(z-z_{d}\right)$.

Within our CEMD model [19], for monochromatic plane wave illumination with wavevector $k_{0}=k_{x}\hat{x}+k_{y}\hat{y}+k_{z}\hat{z}$ and linear polarization v, the field scattered by the metasurface, $\psi_{\mathrm{PW}}^{sca}\left(\omega ,r,k_{0},a_{1},a_{2}\right)$, can be written as:

(6) $$\begin{array}{c} \psi_{\mathrm{PW}}^{sca}\left(\omega ,r,k_{0},a_{1},a_{2}\right)=I\overset{↔}{G}\left(\omega ,r,k_{x},k_{y},a_{1},a_{2}\right)\tilde{\overset{↔}{\alpha }}\left(\omega ,k_{x},k_{y},a_{1},a_{2}\right)v, \end{array}$$

where $a_{1}$ and $a_{2}$ are the primitive vectors that characterize the metasurface. In addition, $\overset{↔}{G}\left(\omega ,r,k_{x},k_{y},a_{1},a_{2}\right)$ is the lattice Green function, and $\tilde{\overset{↔}{\alpha }}\left(\omega ,k_{x},k_{y},a_{1},a_{2}\right)$ is the renormalized polarizability of the metasurface defined by the polarizability of the particles, $\overset{↔}{\alpha }\left(\omega \right)$, and the lattice depolarization Green function, $\overset{↔}{G}\left(\omega ,k_{x},k_{y},a_{1},a_{2}\right)$, as follows:

(7) $$\begin{array}{c} \tilde{\overset{↔}{\alpha }}\left(\omega ,k_{x},k_{y},a_{1},a_{2}\right)=\overset{↔}{\alpha }\left(\omega \right){\left[I-\overset{↔}{G}\left(\omega ,k_{x},k_{y},a_{1},a_{2}\right)\overset{↔}{\alpha }\left(\omega \right)\right]}^{-1}. \end{array}$$

Therefore, the field scattered by the metasurface when illuminated by a point source, $\psi_{d}^{sca}\left(\omega ,r,r_{d}\right)$, can be obtained by replacing the Weyl expression of the incident dipole source, Equation (3), into the corresponding scattered field given by Equation (6), leading to:

(8) $$\begin{array}{ccc} \psi_{d}^{sca}\left(\omega ,r,r_{d}\right) & = & \int dQ_{x}dQ_{y}I^{d}\left(\omega ,r_{d},Q_{x},Q_{y}\right)\overset{↔}{G}\left(\omega ,r,Q_{x},Q_{y}\right)\\ & & \times \tilde{\overset{↔}{\alpha }}\left(\omega ,Q_{x},Q_{y}\right)v^{d}\left(\omega ,Q_{x},Q_{y}\right), \end{array}$$

where the dependencies on primitive vectors, $a_{1}$ and $a_{2}$, have been omitted. Recall that such electromagnetic field ${\psi^{\mathrm{sca}}}_{d}\left(\omega ,r,r_{d}\right)$ is formally equivalent to the Green function of the scattering geometry with argument $r-r_{d}$ within the dipole approximation. Incidentally, we do not refer to it as $\overset{↔}{G}$ in order to avoid confusion with lattice Green functions within our CEMD model. Interestingly, note also that the LDOS of the metasurface (within the dipolar approximation) can be extracted from $\psi_{d}^{sca}\left(\omega ,r,r_{d}\right)$ evaluated at the position of the dipole $r=r_{d}$, as follows:

(9) LDOS(ω,rd)=1+k6πImP†ψdsca(ω,r=rd).

The Green function given by Equation (8), including the LDOS expression (9) are our main analytical results herein, allowing us, in turn, to determine the electromagnetic field close to the metasurface (near field and LDOS) upon point source illumination, indeed crucial to unveil the excitation in the near field of resonant modes and BICs.

Since the Weyl expansion allows us to differentiate between propagating and evanescent components of the dipolar source, it is convenient to split the integral in *Q*-space over $Q_{x},Q_{y}$ in the form:

(10) $$\begin{array}{ccc} \psi^{sca}\left(\omega ,r,r_{d}\right) & = & \int_{Q_{x}^{2}+Q_{y}^{2}<k^{2}}dQ_{x}dQ_{y}I^{d}\left(\omega ,r_{d},Q_{x},Q_{y}\right)\overset{↔}{G}\left(\omega ,r,Q_{x},Q_{y}\right)\\ & & \times \tilde{\overset{↔}{\alpha }}\left(\omega ,Q_{x},Q_{y}\right)v^{d}\left(\omega ,Q_{x},Q_{y}\right)\\ & + & \int_{Q_{x}^{2}+Q_{y}^{2}>k^{2}}dQ_{x}dQ_{y}I^{d}\left(\omega ,r_{d},Q_{x},Q_{y}\right)\overset{↔}{G}\left(\omega ,r,Q_{x},Q_{y}\right)\\ & & \times \tilde{\overset{↔}{\alpha }}\left(\omega ,Q_{x},Q_{y}\right)v^{d}\left(\omega ,Q_{x},Q_{y}\right)\\ & = & \psi_{pro}^{sca}\left(\omega ,r,r_{d}\right)+\psi_{eva}^{sca}\left(\omega ,r,r_{d}\right). \end{array}$$

where the subscripts pro and eva stand for propagating and evanescent waves, respectively. Therefore, it is possible to separately study the contributions of propagating and evanescent waves to the near field, and by extension, to the LDOS. This will be indeed crucial to unveil the evanescent character of BICs.

As a final remark, note that the dipole intensity diverges at $Q_{x}^{2}+Q_{y}^{2}=k^{2}$, so the integral in the Weyl expansion is interpreted as an improper integral. Although well defined, the numeric integration can present problems. To avoid them, a change of variable from Cartesian coordinates ($Q_{x}$ and $Q_{y}$) to pseudo-polar coordinates ($\sqrt{k^{2}\pm Q^{2}}$ and θ) is used. For the propagating components, $Q_{x}^{2}+Q_{y}^{2}<k^{2}$:

(11) $$\begin{array}{ccc} Q_{x} & \to & \sqrt{k^{2}-Q^{2}}\cos\theta \\ Q_{y} & \to & \sqrt{k^{2}-Q^{2}}\sin\theta , \end{array}$$

with 0≤Q<k and −π≤θ<π. On the other hand, for the evanescent components, $Q_{x}^{2}+Q_{y}^{2}>k^{2}$:

(12) $$\begin{array}{ccc} Q_{x} & \to & \sqrt{k^{2}+Q^{2}}\cos\theta \\ Q_{y} & \to & \sqrt{k^{2}+Q^{2}}\sin\theta , \end{array}$$

with 0<Q<∞ and −π≤θ<π. Since the Jacobian of the transformation is proportional to *Q*, the divergence disappears.

## 3. Results and Discussion

### 3.1. Si Nanosphere Metasurface

Let us apply our formulation to explore the LDOS and near field patterns of canonical BICs arising in a metasurface consisting of a square array of Si nanospheres of radius R=100 nm with lattice constant a=b=4R=400 nm. A dispersionless dielectric permittivity of ϵ=12.25 is used to simplify the theoretical analysis, bearing in mind both that it is actually similar to that of Si and other semiconductors (n=3.5), and that losses might be very small for wavelengths above λ∼500 nm (as considered here); see, e.g., Ref. [48] for polycrystalline Si, GaP, etc. The reflectance spectra for varying angles of incidence, calculated as in Ref. [19], are shown in Figure 1b–e. We focus on two symmetry-protected BICs arising at the Γ point from vertical (out-of-plane) dipole resonances (magnetic, MD, for TE polarization at λ=709 nm in Figure 1b, and electric, ED, for TM polarization at λ=552 nm in Figure 1e), which exhibit the expected quasi-BIC bands at off-normal angles with diverging Q-factors that become inaccessible from the far-field exactly at the BIC condition at $\theta =0^{\circ }$.

### 3.2. Vertical Electric-Dipole BIC

**Figure 2:**
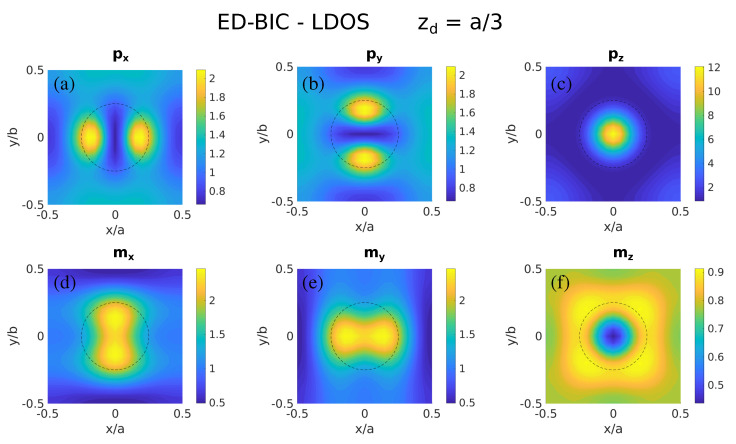
Color maps of the local density of states (LDOS) for the Si nanosphere metasurface shown in Figure 1a, at the wavelength of the symmetry-protected ED-BIC (λ=552 nm; see Figure 1e) within one unit cell at a distance of $z_{d}=a/3\approx 133$ nm, corresponding to the different electromagnetic dipolar contributions: (**a**) $p_{x}$, (**b**) $p_{y}$, (**c**) $p_{z}$, (**d**) $m_{x}$, (**e**) $m_{y}$, (**f**) $m_{z}$. The projection of the nanosphere cross section on the plane is denoted by a dashed circumference.

We first explore in Figure 2 the LDOS for the symmetry-protected ED-BIC shown in Figure 1e at λ=552 nm, on a plane parallel to the metasurface in a region equivalent to one unit cell located at a subwavelength distance $z_{d}=a/3\approx 133$ nm ($z_{d}∼\lambda /4$). All the different electromagnetic contributions are displayed of either electric or magnetic dipolar character along the three Cartesian components ($p_{x}$, $p_{y}$, $p_{z}$, $m_{x}$, $m_{y}$, $m_{z}$). Indeed, even though our formulation only retains the dipolar response of the nanospheres, such contributions correlate quite reasonably with the ED-resonant character of this BIC (basically dipolar indeed): the larger contribution to the LDOS by far is the vertical electric one, $p_{z}$, in turn concentrated at the sphere center, since the ED resonance actually emerges from the electric field pointing perpendicular to the metasurface plane. The other electric in-plane electric contributions, though clearly observable, are substantially weaker, and so are all the magnetic terms. Interestingly, the magnetic vertical dipole $m_{z}$ exhibits a suppression of the LDOS in the entire unit cell.

**Figure 3:**
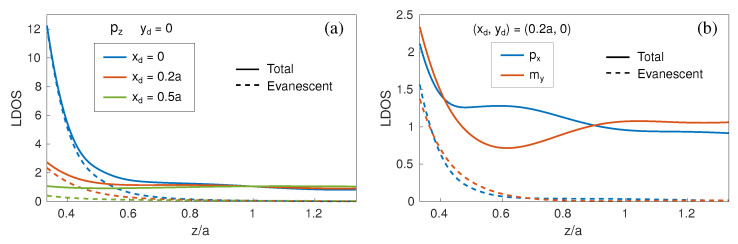
LDOS as a function of the distance from the metasurface plane $z_{d}$ (solid curves) for the Si nanosphere metasurface as in Figure 2, at the wavelength of the symmetry-protected ED-BIC (λ=552 nm), including separately the evanescent contributions (dashed curves): (**a**) $p_{z}$ at $x_{d}=0$ (blue curves), $x_{d}=80$ nm (red curves), and $x_{d}=200$ nm (green curves); (**b**) $p_{x}$ (blue curves) and $m_{y}$ (red curves) at $x_{d}=80$ nm; all for $y_{d}=0$.

We next show in Figure 3a the dependence of the LDOS on the distance to the metasurface $z_{d}$, with $\left(x_{d},y_{d}\right)=\left(0,0\right)$ where the LDOS is maximum (see Figure 2c), including explicitly the evanescent contribution; the limit z→0 is avoided for the sake of validity of our dipolar approach. As the distance becomes smaller than $z_{d}/a∼0.6$ ($z_{d}∼\lambda ∼240$ nm), the contribution to $p_{z}$ from the evanescent components grows exponentially, indicating that a strongly confined, evanescent mode (ED-BIC) is responsible for this enhanced LDOS. It is also evident in Figure 3a that if the $p_{z}$ dipole is displaced from the unit cell center to a position $x_{d}=0.2a=80$ nm where the LDOS decreases, the enhanced evanescent contribution to the LDOS diminishes, in turn becoming nearly negligible at a position $x_{d}=0.5a=200$ nm in the border of the unit cell where the $p_{z}$-LDOS is minimum in Figure 2c. All this evidence supports the fact that such a strongly confined, evanescent mode corresponds to the expected ED-BIC. In addition, we show in Figure 3b the LDOS associated to two in-plane terms, $p_{x}$ and $m_{y}$, at a position where their LDOS is close to the corresponding maxima (see Figure 2a,e). By contrast, these contributions show a small increase of the evanescent components upon approaching the metasurface plane, much weaker than that shown above for the ED-BIC, very likely stemming from the broad in-plane resonances overlapping with the ED-BIC as observed in Figure 1e.

**Figure 4:**
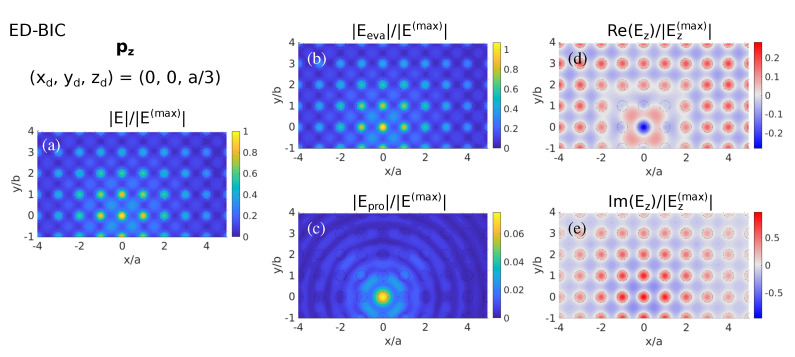
Color maps of the normalized electric near-field on a plane at z=a/3≈133 nm scattered from a Si nanosphere metasurface as in Figure 1, when illuminated by a vertical point ED source at the wavelength of the symmetry-protected ED-BIC (λ=552 nm) located at $\left(x_{d},y_{d},z_{d}\right)=\left(0,0,a/3\right)$; thus, $z_{d}=z\approx 133$ nm. (**a**) Total electric field amplitude, showing separately the contributions from the (**b**) evanescent and (**c**) propagating components, and the real (**d**) and imaginary (**e**) parts of the vertical electric field component.

On the basis of the information provided by the LDOS, we now explore the near-field (NF) excitation of the BIC. According to Figure 2c and Figure 3a, we place a vertical ED source at $r_{d}=\left(0,0,a/3\right)$ (thus, $z_{d}\approx 133$ nm), where the $p_{z}$-LDOS is very large. The resulting near-field map on a parallel plane at a fixed distance $z=z_{d}\approx 133$ nm from the metasurface is shown in Figure 4, separated into components. The total electric field amplitude in Figure 4a reveals a slightly higher concentration close to the projection of the ED source (center of the Si nanosphere metasurface), but, most importantly, the electric NF spreads over the metasurface plane without apparent decay. Indeed, if we analyze separately the contributions from the evanescent and propagating components (see Figure 4b,c, respectively), we observe that the propagating components radiate away from the metasurface center, wherein the dipole source is located, emerging as outgoing spherical waves with decaying amplitude. Conversely, we clearly identify the evanescent components in Figure 4b as responsible for the non-leaky mode across the metasurface plane expected for a BIC. Moreover, if we focus on the $E_{z}$ component shown in Figure 4d,e, we confirm the expected NF pattern of the corresponding vertical-ED BIC, spreading with identical phase in every unit cell, thus precluding radiation by symmetry protection. This, in turn, supports the correlation between this planar constant NF pattern in Figure 4a,b and the enhanced (evanescent) $p_{z}$-LDOS in Figure 2c with the symmetry-protected ED-BIC.

**Figure 5:**
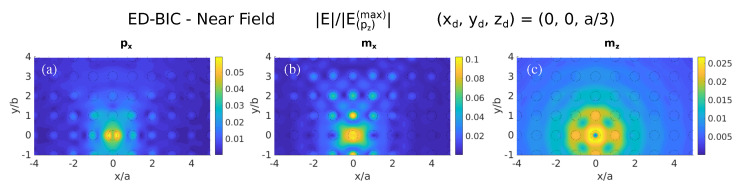
Color maps of total electric near-fields as in Figure 4a at the wavelength of the symmetry-protected ED-BIC (λ=532 nm), but illuminating with various point-dipole sources located also at $\left(x_{d},y_{d},z_{d}\right)=\left(0,0,a/3\right)$: (**a**) in-plane electric ($p_{x}$); (**b**) in-plane magnetic ($m_{x}$); and (**c**) vertical magnetic ($m_{z}$). In all cases, the electric field is normalized for the sake of comparison to the maximum of the field when excited by a vertical electric dipole $p_{z}$.

To further confirm such correlation, we next show in Figure 5 the NF maps arising with other point-dipole sources located also at $z_{d}=z=a/3\approx 133$ nm: in-plane electric ($p_{x}$) and magnetic ($m_{x}$), and vertical magnetic ($m_{z}$). Only in the unit cells corresponding to the location of the dipole source and nearest neighbors the NF pattern is significant, indicating that the electromagnetic field is basically scattered away from the metasurface, thus showing negligible excitation of the ED-BIC. In addition, it can be seen that the fields present much lower intensity than those arising when excited by the vertical electric dipole ($p_{z}$, Figure 2). It is evident that none of them are able to efficiently couple to the ED-BIC, as predicted by the low LDOS stemming from such components; see Figure 2a,d,f.

**Figure 6:**
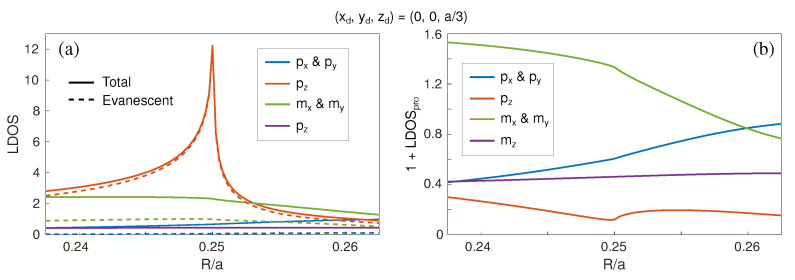
LDOS for the Si nanosphere metasurface shown in Figure 1a as a function of R/a for a fixed wavelength (λ=552 nm) at the unit cell center and at distance of $z_{d}=a/3\approx 133$ nm. All the different electromagnetic dipolar contributions are included: in-plane electric ($p_{x},p_{y}$, blue) and magnetic ($m_{x},m_{y}$, green), and vertical electric ($p_{z}$, red) and magnetic ($m_{z}$, purple), separating: (**a**) total (solid curves) and evanescent (dashed curves) contributions; and (**b**) propagating contributions. The maximum of the evanescent $p_{z}$ contribution at R/a∼0.25 corresponds to the wavelength of the symmetry-protected ED-BIC at the Γ point in Figure 1e for R=100 nm.

Finally, we also consider in Figure 6 the LDOS at the wavelength of the ED-BIC (λ=552 nm) as a function of the nanosphere radius for all vectorial components. Clearly, the evanescent contribution to the $p_{z}$ term in Figure 6a shows a large LDOS maximum at the ED-BIC condition R/a=1/4, which abruptly decreases with a slight mismatch in nanosphere size. Recall that this variation is nearly equivalent to a frequency mismatch for fixed nanosphere radius, so that this peak qualitatively correlates with the diverging Q-factor expected upon approaching the BIC, only limited here by the fact that the LDOS at a given distance from the metasurface remains finite. As expected, the other evanescent contribution to all other LDOS terms in Figure 6a remain structureless (and nearly negligible, except for a small contribution from the in-plane magnetic terms), and so do all the propagating contributions in Figure 6b.

### 3.3. Vertical Magnetic-Dipole BIC

**Figure 7:**
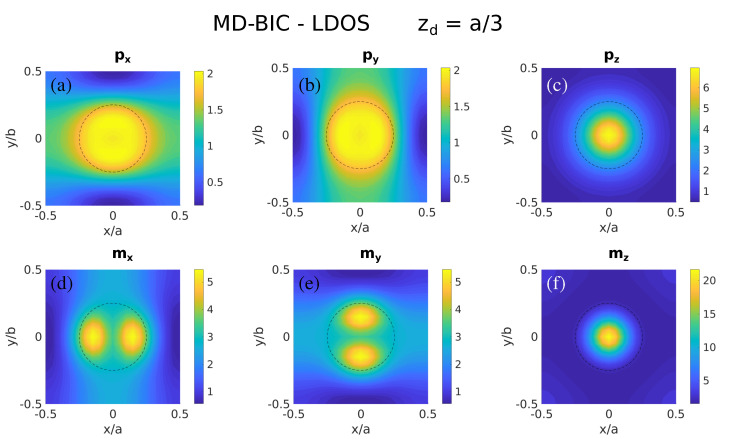
Color maps of the LDOS as in Figure 2, but at the wavelength of the symmetry-protected MD-BIC (λ=709 nm and $z_{d}=a/3\approx 133\;\mathrm{nm}∼0.19\lambda$): (**a**) $p_{x}$, (**b**) $p_{y}$, (**c**) $p_{z}$, (**d**) $m_{x}$, (**e**) $m_{y}$, (**f**) $m_{z}$.

For the sake of completeness, let us analyze the LDOS of the other symmetry-protected BIC supported by the Si nanosphere metasurface as shown in Figure 1b. The resulting LDOS maps are presented in Figure 7. First, the MD-BIC is neatly evident in Figure 7f through a large contribution from the vertical MD, $m_{z}$, concentrated as expected at the vertical of the sphere center. Other moderately strong in-plane electric contributions are obtained (see Figure 7a,b), since the MD resonance actually emerges from the electric field circulating inside the nanospheres (recall that the Si nanospheres are non-magnetic). Interestingly, note also that the in-plane $m_{x},m_{y}$ contributions (and also as a significant $p_{z}$ term) yield relatively strong LDOS (see Figure 7d,e,c). This stems from the fact that the MD resonance is degenerate for a sphere due to symmetry and the proper MD-BIC coexists with a relatively strong in-plane magnetic contribution revealed as a broad background in Figure 1b. The reason that this degeneracy-induced coexistence is observed for the MD-related BIC, but not for the ED-BIC shown above, most likely stems from the impact of the lattice: weaker (respectively, stronger) in-plane coupling of the vertical MD (respectively, ED) imposes a smaller (respectively, larger) frequency shift of the MD-BIC (respectively, ED) with respect to the bare MD (respectively, ED) nanosphere resonance.

**Figure 8:**
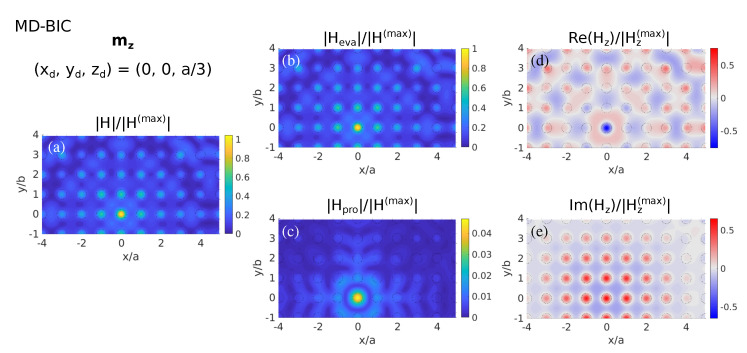
Color maps of near-fields as in Figure 4, but for the magnetic field at the wavelength of the symmetry-protected MD-BIC (λ=709 nm), illuminating with a vertical point MD source located at $\left(x_{d},y_{d},z_{d}\right)=\left(0,0,a/3\right)$; thus, $z_{d}=z\approx 133$ nm: (**a**) Total magnetic field amplitude, showing separately the contributions from the (**b**) evanescent and (**c**) propagating components, and the real (**d**) and imaginary (**e**) parts of the vertical electric field component.

Let us next explore how the LDOS relates to the NF excitation shown in Figure 8. As expected, it is evident in Figure 8a that the vertical MD source ($m_{z}$) excites the MD-BIC spreading without losses across the metasurface plane. Indeed, this MD-BIC behavior stems from the evanescent contribution (see Figure 8b), with the propagating contribution revealing radiation away from the unit cell close to the MD source (see Figure 8c). Furthermore, the vertical magnetic field component is shown to be the predominant one, and its phase-matched condition across unit cells corroborates the symmetry-protection mechanism that precludes leakage of this MD-BIC (see Figure 8d,e).

## 4. Conclusions

In summary, we have developed a coupled electric and magnetic dipole analytical formulation to determine the Green function for an infinite planar array of electric/magnetic dipoles. This allows us to obtain any physical magnitude connected to the excitation by a point dipole source: particularly, we have focused on two relevant magnitudes: LDOS and near fields. Those magnitudes are of the utmost importance when dealing with so-called BICs, since these modes cannot be probed by definition from the far field (except indirectly). Thereby, we have investigated the LDOS associated to symmetry-protected BICs emerging in a metasurface consisting of an square array of Si nanospheres. The LDOS at a sub-wavelength distance from the metasurface clearly describe the nature of such BICs (vertical ED or MD, both with predominant evanescent contributions), in turn revealing the most appropriate location over the unit cell to effectively couple to the corresponding BIC mode. Such information is used to calculate the near field pattern when the metasurface is excited by a suitable point dipole source, leading to BIC excitation across the metasurface without leakage; the resulting pattern indeed supports their symmetry-protection mechanisms. Our formulation can be exploited to investigate BICs and any other guided/leaky modes supported by metasurfaces and metagratings of interest throughout the electromagnetic spectrum, as long as the predominant optical response of the meta-atoms is of electric and/or magnetic dipolar nature, which is the case of many plasmonic, all-dielectric, and/or hybrid sub-wavelength scatterers. In this regard, recall that our coupled electric/magnetic dipole model for far-field illumination has proven very accurate, not only qualitatively but even quantitatively, in describing the properties of a variety of metasurfaces in the microwave, THz, and visible domains [46,47,48,59,60]. Moreover, cutting-edge experiments for near-field excitation based on the model presented here are indeed underway.
